# Supplementary material for: Identifying and prioritising barriers to injury care in Northern Malawi, results of a multifacility multidisciplinary health facility staff survey
Source: PLoS One. 2024 Sep 12;19(9):e0308525. doi: 10.1371/journal.pone.0308525 (PMC11392338; doi:10.1371/journal.pone.0308525)
Supplement: S2 File — 1) List of barriers healthcare worker participants could select from, by delay, 2) Breakdown of other category of healthcare worker, 3) Other barriers proposed in the healthcare worker survey and their corresponding prioritisation, 4) Table demonstrating frequency of barriers reported by health facility staff within the top 3 most important overall according to participant place of work (primary or referral facility). (PDF) [file pone.0308525.s002.pdf]

Supplementary Tables

S1 Table List of barriers healthcare worker participants could select from, by Delay.

**Delay 1 (Seeking Care)**
The perceived financial costs associated with seeking care are too great
Normal cultural behaviours delay seeking care such as gender roles, family responsibilities and requiring someone else's permission to seek care
People perceive that care is too difficult to physically access
People don't understand about health or available healthcare.
People perceive that available facility care is poor quality
There are delays in discovering injured people, including because of intoxication
People prefer traditional healers
People fear the consequences of helping an injured person, e.g. being accused of causing the injury*
**Delay 2 (Reaching Care)**
There is a lack of timely affordable emergency transport (formal or informal)
There is a large physical distance from place of injury to an appropriate healthcare facility
There is a lack of timely available prehospital emergency care (formal or informal/bystander)
There is a lack of accessible emergency assistance communication mechanism (e.g. emergency call centre)
There is a lack of emergency care service coordination, including bypassing unsuitable facilities or transferring between facilities
There is a lack of reliable uncongested roads with priority for emergency vehicles
**Delay 3 (Receiving Care)**
There is a lack of reliably available necessary physical resources (e.g. infrastructure, equipment and consumable material)
In regard to staffing, there is a lack of reliably available, suitably trained and motivated clinical staff
Specialist services needed for some injuries are not available in this area.
There is a lack of good quality, structured care processes for injured patients.
Lack of available means to safely and quickly transfer injured patients on to a more specialist facility*
In regard to patient demand, there is insufficient facility capacity to meet patient demand (e.g. overcrowding)
There is a lack of patient and family cooperation with care processes
Difficulties with timely payment for care
Need for unauthorised payments or gifts to healthcare staff to receive best available treatment. (e.g. corruption) *
Footnote - *Barrier included after identification from concurrent community-based study

S2 Table Breakdown of other category of healthcare worker.

Other healthcare workers		102 (44.7)
	Other - Administrator	4 (1.8)
	Other - Allied Health Professional	14 (6.1)
	Other - Ambulance Driver	10 (4.4)
	Other - Community Health Worker	10 (4.4)
	Other - Ground labourer	3 (1.3)
	Other - Patient Attendant*	6 (2.6)
	Other - Security	15 (6.6)
	Other - Ward or Hospital attendant of equivalent**	40 (17.5)
*Patient attendants are trained on the job to perform relatively basic clinical tasks such as observations, wound dressing and suturing wounds. **Ward or Hospital attendants provide primarily domestic function although may perform some clinical tasks such as wound dressing.		

S3 Table - Other barriers proposed in the healthcare worker survey and their corresponding prioritisation.

Barrier	Explanation	Possible incorporating original barrier
**Delay 1**		
People undermine the severity of the injury	Many delay seeking care after they experience additional infections	People don't understand about health or available healthcare.
People think about the attitude of health workers prior to decide when to seek help.	Many people fail to decide to go to the health facility earlier because they fear the health workers will not assist them in time. Especially worse during weekends	People perceive that available facility care is poor quality
Distance between household and their own initial assessment of the degrees of the injuries	Some household are too far apart in some areas, that calling for assistance or help in times of injuries is so difficult. Many of the patients or relatives tend to do initial assessment on the degrees of the injury to the patient which usually do not bear the real clinical assessment.	People perceive that care is too difficult to physically access
Decision making as to regards to the degrees of injuries after self-assessment.	These usually delays the injured patient accessing care in time.	People don't understand about health or available healthcare.
Altitude of health workers	These affects the injured patients to seek care earlier than normal because they think first how health workers will receive them.	People perceive that available facility care is poor quality
If it is a gender-based violence	Partners takes time to report in fear of harming their spouses or making them arrested.	People fear the consequences of helping an injured person
People fear of infections	There is a fear of people contracting infections such as HIV and Hepatitis, since many don't have protective wear readily available in their homes.	People perceive that available facility care is poor quality
Fear of the law by the individuals which themselves might have caused.		People fear the consequences of helping an injured person
Proximity of other health facilities	These usually reduce the level of efficiency for a tertiary level hospital.	People perceive that care is too difficult to physically access
**Delay 2**		
In case of hit and run road accident in the night many people are discovered in the morning while injured and intoxicated.	Many accidents do happen during the night and those injured are at difficult to find help.	There are delays in discovering injured people, including because of intoxication
Use of untrained ways of carrying injured patients to the clinic.	These usually aggregates the condition of patients e.g. with spinal injury.	There is a lack of timely available prehospital emergency care (formal or informal/bystander)
Inaccessible roads	Places like chibalabala near the park	There is a lack of reliable uncongested roads with priority for emergency vehicles
Lack of fuel in emergency vehicles	These delays the available vehicles reach the scene of accident late than expected.	There is a lack of timely affordable emergency transport (formal or informal)
Hide of information by injured people denies them access to ways to reach hospital earlier.	Bystanders or medical fail to help the injured in time due to insufficient information provided some injured people.	People don't understand about health or available healthcare.
Government policy should introduce in the driving school module about first aid.	These will enhance first primary care in the communities.	There is a lack of timely available prehospital emergency care (formal or informal/bystander)
**Delay 3**		

People are sent back to police to collect statement of injury	It causes delay since people are left unattended until the report is sent from the police	NOVEL
Most of the time untrained staff attend to injured persons	People receive inadequate care because most of the time those on duty are not medically trained to treat those injured. There is a need of seriousness on healthy workers or those assigned on duty.	In regard to staffing, there is a lack of reliably available, suitably trained and motivated clinical staff
Lack of proper emergency care room at the facility with readily available trained staff.	Especially during the weekends when staffing levels at the hospital is inadequate, it takes time to call for assistance for trained personnel.	There is a lack of reliably available necessary physical resources (e.g. infrastructure, equipment and consumable material) AND In regard to staffing, there is a lack of reliably available, suitably trained and motivated clinical staff
Professional policy or workplace policy that governs the management of the injured patients.	In other way the bureaucracy that is in place in most institution trusts the development of motivated staff or work facility management.	There is a lack of good quality, structured care processes for injured patients.
Inconsistent power supply from Escom	Patients wait for so long to get x-rayed or operations done.	There is a lack of reliably available necessary physical resources (e.g. infrastructure, equipment and consumable material)
Work schedule between staff members and current hospital referral system	Lack of coordination between members of staff during work shifts usually vacuum to unattended work posts.	In regard to staffing, there is a lack of reliably available, suitably trained and motivated clinical staff
Special people should be assigned to trauma cases	These will help unnecessary delay to patient care as of now many cases are delayed because of shouldering responsibilities.	In regard to staffing, there is a lack of reliably available, suitably trained and motivated clinical staff AND There is a lack of good quality, structured care processes for injured patients.
In-house refresher trainings for situation like mass casualties	Most of the time are called to the casualty department yet they haven't had or did have little training in the care of the injured people.	In regard to staffing, there is a lack of reliably available, suitably trained and motivated clinical staff
Treatment missed guideline that some patients are wrongly diagnosed at first.	These patients usually	There is a lack of good quality, structured care processes for injured patients.
Missed during triage as an emergency and treated as outpatient care.	Patient are sent home while proper investigation has not been conducted. These might be due to clinicians negligence or electricity blackout or malfunction machines.	There is a lack of good quality, structured care processes for injured patients.
Creation of strategic trauma centres along the M1 ROAD or major trading centres	Copy from other countries how they manage the situation with trauma centres. These will greatly reduce the fatality cases and care of trauma care patient.	Specialist services needed for some injuries are not available in this area.
Poor attitudes of health workers and negligence ranked at 1	Domestic violence victims are not well assisted, so next time they are abused again they delay seeking care	In regard to staffing, there is a lack of reliably available, suitably trained and motivated clinical staff

S4 Table demonstrating Frequency of barriers reported by health facility staff within the top 3 most important overall according to participant place of work (primary or referral facility)

Barrier	Primary (104 participants)	Referral (124 participants)
There is a lack of reliably available necessary physical resources (e.g. infrastructure, equipment and consumable material)	35	60
In regards to staffing, there is a lack of reliably available, suitably trained and motivated clinical staff	55	34
There is a lack of timely affordable emergency transport (formal or informal)	32	36
Lack of available means to safely and quickly transfer injured patients on to a more specialist facility.	23	26
Specialist services needed for some injuries are not available in this area	19	24
The perceived financial costs associated with seeking care are too great	30	8
There is a large physical distance from place of injury to an appropriate healthcare facility	16	15
There is a lack of accessible emergency assistance communication mechanism (e.g. emergency call centre)	14	15
People don't understand about health or available healthcare.	17	9
There is a lack of timely available pre hospital emergency care (formal or informal/bystander)	9	16
In regards to patient demand, there is insufficient facility capacity to meet patient demand (e.g. overcrowding)	6	14
People prefer traditional healers	7	13
There is a lack of good quality, structured care processes for injured patients.	10	9
Normal cultural behaviours delay seeking care such as gender roles, family responsibilities and requiring someone else's permission to seek care	10	8
There is a lack of patient and family cooperation with care processes	6	9
There is a lack of emergency care service coordination, including bypassing unsuitable facilities or transferring between facilities	9	6
People fear the consequences of helping an injured person, e.g. being accused of causing the injury.	7	7
People perceive that available facility care is poor quality	6	6
There are delays in discovering injured people, including because of intoxication	3	7
Need for unauthorised payments or gifts to health care staff to receive best available treatment. (e.g. corruption)	7	1
There is a lack of reliable uncongested roads with priority for emergency vehicles	5	3
People perceive that care is too difficult to physically access	1	6
Receiving Other	1	3
Difficulties with timely payment for care	2	2
Reaching Other	0	1

Seeking Other	0	0
