# Supplementary material for: Identifying and prioritising barriers to injury care in Northern Malawi, results of a multifacility multidisciplinary health facility staff survey
Source: PLoS One. 2024 Sep 12;19(9):e0308525. doi: 10.1371/journal.pone.0308525 (PMC11392338; doi:10.1371/journal.pone.0308525)
Supplement: Attachment — Submitted filename: RTR HCW PLOSONE v1.2.docx [file pone.0308525.s003.docx]

Dear Editor

We are pleased to respond to the editor and reviewer comments to improve our manuscript. Please find our responses inline below.

On behalf of the authors

Journal Requirements:

When submitting your revision, we need you to address these additional requirements.

1. Please ensure that your manuscript meets PLOS ONE's style requirements, including those for file naming. The PLOS ONE style templates can be found at 
<https://journals.plos.org/plosone/s/file?id=wjVg/PLOSOne_formatting_sample_main_body.pdf> and 
<https://journals.plos.org/plosone/s/file?id=ba62/PLOSOne_formatting_sample_title_authors_affiliations.pdf>

We have renamed figure and supplementary files accordingly.

2. Thank you for stating the following financial disclosure: 
"This work was supported by a research fellowship awarded to JW from the Royal
College of Surgeons of England and The King’s Centre for Global Health and Health
Partnerships."
 
Please state what role the funders took in the study.  If the funders had no role, please state: ""The funders had no role in study design, data collection and analysis, decision to publish, or preparation of the manuscript."" 
If this statement is not correct you must amend it as needed. 
Please include this amended Role of Funder statement in your cover letter; we will change the online submission form on your behalf.

Thank you for changing the online submission for our amended financial disclosure which should read as follows:

“This work was supported by a research fellowship awarded to JW from the Royal
College of Surgeons of England and The King’s Centre for Global Health and Health
Partnerships. The funders had no role in study design, data collection and analysis, decision to publish, or preparation of the manuscript.”

3. We note that you have indicated that there are restrictions to data sharing for this study. For studies involving human research participant data or other sensitive data, we encourage authors to share de-identified or anonymized data. However, when data cannot be publicly shared for ethical reasons, we allow authors to make their data sets available upon request. For information on unacceptable data access restrictions, please see <http://journals.plos.org/plosone/s/data-availability#loc-unacceptable-data-access-restrictions>.

Before we proceed with your manuscript, please address the following prompts:

a) If there are ethical or legal restrictions on sharing a de-identified data set, please explain them in detail (e.g., data contain potentially identifying or sensitive patient information, data are owned by a third-party organization, etc.) and who has imposed them (e.g., a Research Ethics Committee or Institutional Review Board, etc.). Please also provide contact information for a data access committee, ethics committee, or other institutional body to which data requests may be sent.

b) If there are no restrictions, please upload the minimal anonymized data set necessary to replicate your study findings to a stable, public repository and provide us with the relevant URLs, DOIs, or accession numbers. Please see <http://www.bmj.com/content/340/bmj.c181.long> for guidelines on how to de-identify and prepare clinical data for publication. For a list of recommended repositories, please see <https://journals.plos.org/plosone/s/recommended-repositories>. You also have the option of uploading the data as Supporting Information files, but we would recommend depositing data directly to a data repository if possible.

Please update your Data Availability statement in the submission form accordingly.

We have updated the data availability statement as follows:

Data cannot be shared openly due to risks of identifying healthcare workers. However, they can be made available from Malawi Epidemiology and Intervention Research Unit (MEIRU) on reasonable request and following signing of a data transfer agreement. Data enquiries should be sent to [info@meiru.mw](mailto:info@meiru.mw), quoting the title of the paper.

Reviewers' comments:

Reviewer's Responses to Questions

**Comments to the Author**

Review Comments to the Author

Reviewer #1: The authors report on the results of a survey of health care workers, primarily, from several health care centers in Malawi. A total of 228 workers, mostly clinicians, were asked about barriers that lead to delays in either seeking, reaching, or receiving injury (trauma) care. Overall, the manuscript is very well written with minimal need for editing (see below single comment). However, the results are not surprising, given the population interviewed, and mirror those of other surveys from sub-Saharan Africa. As mentioned by the authors, there are key differences between centers that likely influence the prioritization given to the questions posed. For example, the faith-based facilities required payment for services, making this a much more significant barrier for patients, but could lead to better resource availability due to reduced demand and higher revenue. As a result of pooling all the responses together, the authors likely suppressed any key differences in barriers to care seeking and delivery that may exist between care delivery models. The analysis would be more informative if it was analyzed by site type. Public vs Military vs Private (faith-based). Or if that is not possible, at least by level, so that responses at the tertiary facility are looked at separate from the “front line” facilities.

Thank you for the comments. Comparative analysis was not the main objective of this paper which was to more broadly synthesise the views of health facility staff around the relative importance of different health system barriers. Since the majority of health facility staff included worked in public facilities, comparison by specific facility type may be less useful. We have, however, tabulated the barriers considered the top 3 most important overall according to whether the participant worked in a referral (secondary or tertiary) or a primary facility. We have described this in the discussion as per the below text as an exploratory analysis and provided the analysis within a supplementary table.

“As an exploratory analysis, we considered whether staff in referral (secondary or tertiary) facilities prioritised different barriers from the primary facility staff. This is shown in S4 Table and shows that both primary and referral facility staff listed similar barriers amongst the top 3 most important. Interestingly, whilst both physical resources and staffing were amongst the top 3 barriers for both referral and primary facility staff, referral facility staff more commonly prioritised physical resources whereas for in primary facilities staffing issue were more commonly prioritised, perhaps due to primary facility staff often working alone or in small numbers (1). Furthermore, for primary facility participants, costs for seeking care and healthcare literacy were also relatively more often prioritised, perhaps related to their proximity to the communities where these issues are understood to prevent further care in referral facilities. Insufficient facility capacity was more commonly prioritised by referral facility staff, possibly related to a concentration of more unwell patients not able to be managed by primary facilities (2). A lack of prehospital care was also relatively more often prioritised by referral facility staff, who perhaps more commonly experienced the consequences of patients who might have benefited from earlier therapeutic intervention on route to the facility (3).”

**S4 Table demonstrating Frequency of barriers reported by health facility staff within the top 3 most important overall according to participant place of work (primary or referral facility)**

| **Barrier** | **Primary (104 participants)** | **Referral (124 participants)** |
| --- | --- | --- |
| There is a lack of reliably available necessary physical resources (e.g. infrastructure, equipment and consumable material) | 35 | 60 |
| In regards to staffing, there is a lack of reliably available, suitably trained and motivated clinical staff | 55 | 34 |
| There is a lack of timely affordable emergency transport (formal or informal) | 32 | 36 |
| Lack of available means to safely and quickly transfer injured patients on to a more specialist facility. | 23 | 26 |
| Specialist services needed for some injuries are not available in this area | 19 | 24 |
| The perceived financial costs associated with seeking care are too great | 30 | 8 |
| There is a large physical distance from place of injury to an appropriate healthcare facility | 16 | 15 |
| There is a lack of accessible emergency assistance communication mechanism (e.g. emergency call centre) | 14 | 15 |
| People don't understand about health or available healthcare. | 17 | 9 |
| There is a lack of timely available pre hospital emergency care (formal or informal/bystander) | 9 | 16 |
| In regards to patient demand, there is insufficient facility capacity to meet patient demand (e.g. overcrowding) | 6 | 14 |
| People prefer traditional healers | 7 | 13 |
| There is a lack of good quality, structured care processes for injured patients. | 10 | 9 |
| Normal cultural behaviours delay seeking care such as gender roles, family responsibilities and requiring someone else's permission to seek care | 10 | 8 |
| There is a lack of patient and family cooperation with care processes | 6 | 9 |
| There is a lack of emergency care service coordination, including bypassing unsuitable facilities or transferring between facilities | 9 | 6 |
| People fear the consequences of helping an injured person, e.g. being accused of causing the injury. | 7 | 7 |
| People perceive that available facility care is poor quality | 6 | 6 |
| There are delays in discovering injured people, including because of intoxication | 3 | 7 |
| Need for unauthorised payments or gifts to health care staff to receive best available treatment. (e.g. corruption) | 7 | 1 |
| There is a lack of reliable uncongested roads with priority for emergency vehicles | 5 | 3 |
| People perceive that care is too difficult to physically access | 1 | 6 |
| Receiving Other | 1 | 3 |
| Difficulties with timely payment for care | 2 | 2 |
| Reaching Other | 0 | 1 |
| Seeking Other | 0 | 0 |

While the perspectives of the care providers were argued to also reflect the views of the patients in the communities served, this is not necessarily true. Why weren’t patients surveyed? Were they not also in the facilities that were visited? Having both perspectives and looking at areas of alignment would have been more useful in planning and design of future interventions.

We have added to our discussion this limitation as follows:

“Our study focused on health facility staff perspectives and did not have a patient voice. There are potential methodological, logistical, and ethical challenges to concurrently surveying patients during a time limited facility visit. For many of the primary facilities, there may not have been an injured patient present during the facility visit. At larger facilities, surveying patients during acute care has practical and ethical difficulties depending upon the nature of their injuries. We have previously used alternative methodological approaches such as community focus groups and household surveys to obtain a patient perspective complementary to the facility staff perspectives this study has captured.(4, 5)”

It is human nature to say that you could do a better job if you had more resources (even in HICs), so this too is a significant bias affecting the survey results. While it is simple to say the facilities lack human and supply resources and this is perceived as easily addressable, the reality of creating a sustainable change in any health care related resource is far from simple. Thus, it is of interest to know how these providers felt this could be easily achieved? What is it they needed that was easily implemented? Again, this is key to making the reported information useful in future efforts to improve care in the region.

This is an important point but a limitation of our study in that we did not explore what interventions the participants thought would be useful to address the barriers to care they perceived. This is potentially a limitation of the three delays approach to health system evaluation which has a problem focussed nature rather than a solutions focussed nature. We have added this point to the limitations discussion.

“However, the Three Delays framework can be limited as being barrier or problem focussed rather than prompting identification or delivery of solutions.(6) In our study we have not explored the participants’ ideas as to what should be done to address the barriers highlighted which could be an important avenue for further research.”

From the data presented in figure 6, the lack of transport to a facility for care stands out, with multiple orange bars essentially highlighting the cost and availability of transport as a major barrier. From the time data, about half seem to get care within 2 hours, but the remaining half face much longer delays, and this is perceived as being very harmful. Thus, the greatest impact on the greatest number would seem to be getting patients to the care that is available, and not necessarily changing the care that is delivered in under an hour for most, based on the data. This conclusion would seem to be at odds with the current conclusions of the manuscript. “Health facility staff priorities should inform health system improvements, although specific findings may differ between health systems and from the priorities of community members and patients.” One could argue, alternatively, in a resource limited environment, the greatest good for the greatest number should, perhaps, drive policy.

We do not think this argument is at odds with the paper’s recommendation. This is an approach to ascertaining a set of stakeholders (Health facility staff) views on where the greatest good could be achieved.

While some of the biases and influences on the results are part of the discussion throughout, the formal limitation paragraph is underdeveloped and should include a concise summary of the biases and limitations of the approach taken. What were limits on analysis? Why did you consolidate so many categories into dichotomous groups? Presumably there were some key limitations to the analyses that were the bases for this and should be addressed. If the above suggested analyses can’t be performed, then this should also be addressed.

We agree that our discussion of limitations to our study is broader than a single paragraph. We have therefore introduced the word limitation earlier in the discussion to highlight what we believe to be a thorough discussion of the limitations of this study. We have further added to the limitations in accordance to our earlier responses. Regarding dichotomous groups, if this refers to the description of receipt of clinical training, both type and how long ago, this was described according to whether or not they were a clinical care provider. This is relevant since those not providing clinical care would be unlikely to undergo advanced injury care training. We have addressed the point about additional analyses earlier.

As presented, the results and discussion do not provide a foundation or direction for future efforts to impact the care delivery in the region. Presumably the purpose for this work was to identify barriers that could be addressed. Thus, consolidating some of the discussion to focus on the key results and the implications for future efforts to impact care would be welcome changes in the manuscript.

We have added the following text to the discussion to further explore implications for future research:

“Whilst our study has demonstrated value in applying a Three Delays framed survey of health facility staff for injury health system evaluation, additional data sources and methods are needed to more fully understand inherently complex and adaptive health systems (7). Also, barriers to accessing quality care may differ between context and countries (8). More rigorous evaluations using mixed methods approaches may be better suited to drive interventions and policy change (5, 8). Future research through the NIHR funded Equi-Injury collaborative will advance a mixed methods Three Delays framed approach to injury care health system assessment in four different LMICs to co-develop improvements in equitable injury care (9).”

One typo noted on page 14: I think you may want to change “meaningful” to “meaningfully”
“…offers an expeditious way to meaningful improve the system of care they work within…”

We have changed this as advised, thank you.

1.	Chokotho L, Mulwafu W, Singini I, Njalale Y, Jacobsen KH. Improving hospital-based trauma care for road traffic injuries in Malawi. World J Emerg Med. 2017;8(2):85-90.

2.	Chokotho L, Mulwafu W, Jacobsen KH, Pandit H, Lavy C. The burden of trauma in four rural district hospitals in Malawi: a retrospective review of medical records. Injury. 2014;45(12):2065-70.

3.	Chokotho L, Mulwafu W, Singini I, Njalale Y, Maliwichi-Senganimalunje L, Jacobsen KH. First Responders and Prehospital Care for Road Traffic Injuries in Malawi. Prehosp Disaster Med. 2017;32(1):14-9.

4.	Whitaker J, Amoah AS, Dube A, Rickard R, Leather AJM, Davies J. Access to quality care after injury in Northern Malawi: results of a household survey. BMC Health Serv Res. 2024;24(1):131.

5.	Whitaker J, Edem I, Togun E, Amoah AS, Dube A, Chirwa L, et al. Health system assessment for access to care after injury in low- or middle-income countries: A mixed methods study from Northern Malawi. PLoS Med. 2024;21(1):e1004344.

6.	Actis Danna V, Bedwell C, Wakasiaka S, Lavender T. Utility of the three-delays model and its potential for supporting a solution-based approach to accessing intrapartum care in low- and middle-income countries. A qualitative evidence synthesis. Glob Health Action. 2020;13(1):1819052.

7.	Whitaker J, Donohoe N, Denning M, Poenaru D, Guadagno E, Leather AJM, Davies JI. Assessing trauma care systems in low-income and middle-income countries: a systematic review and evidence synthesis mapping the Three Delays framework to injury health system assessments. BMJ Glob Health. 2021;6(5):e004324.

8.	Odland ML, Abdul-Latif A-M, Ignatowicz A, Alayande B, Appia Ofori B, Balanikas E, et al. Equitable access to quality trauma systems in low-income and middle-income countries: assessing gaps and developing priorities in Ghana, Rwanda and South Africa. BMJ Glob Health. 2022;7(4):e008256.

9.	Davies J, Chu K, Tabiri S, Byiringiro JC, Bekele A, Razzak J, et al. Equitable access to quality injury care; Equi-Injury project protocol for prioritizing interventions in four low- or middle-income countries: a mixed method study. BMC Health Serv Res. 2024;24(1):429.
