# Supplementary material for: Identifying and prioritising barriers to injury care in Northern Malawi, results of a multifacility multidisciplinary health facility staff survey
Source: PLoS One. 2024 Sep 12;19(9):e0308525. doi: 10.1371/journal.pone.0308525 (PMC11392338; doi:10.1371/journal.pone.0308525)
Supplement: Attachment — Submitted filename: RTR re-revision.docx [file pone.0308525.s004.docx]

RTR re-revision

**Reviewer #1:** The authors have addressed the primary concerns raised by the initial review. A few minor edits should be made prior to publication.

Regarding the figures, Figures 1 and 6 should use the same colours to represent the given delays.

These have been edited to have the same colour scheme.

It is noted that figure titles are provided within the text, presumably as place holders for figure location, but these should also have figure legends to support interpretation of the figure as a stand-alone presentation of the data. Key findings should be noted in the legend and any significant differences upon which statistical analysis was performed should be highlighted. This latter aspect may not be relevant for the figures in this manuscript, but may be relevant to any supplemental data and figures.

We have added figure legends to support interpretation as follows:

**Figure 1 Health facility staff perceptions of importance for each of the Three Delays.** *Whilst delay 3 was not considered by most participants to either affect the largest number of people, nor cause the longest delay, most participants considered it the most amenable to change and the most important delay overall.*

**Figure 2 Health facility staff perceptions of the proportion of patients delaying seeking and receiving care.** *Most participants thought half or more patients delayed seeking care, but few or no patients experienced delays in receiving care.*

**Figure 3 Health facility staff perceptions of time delays to seeking and receiving care.** *Most participants thought delays in patients seeking care were typically 2 hours or longer, whereas most thought delays in receiving care were less than 1 hour.*

**Figure 4 Health facility staff perceptions of patient harm associated with each delay.** *A substantial majority of participants considered patients with delays to seeking care, delays to reaching care of more than 4 hours and delays in receiving care suffered significant or very significant harm.*

**Figure 5 Health facility staff perceptions of the proportion of injured patients reaching care within given time frames from injury.** *The majority of participants thought few injured patients could reach care within 2 hours.*

**Figure 6 Frequency of barriers reported by health facility staff within the top 3 most important overall (colour coded by delay stage).** *Barriers from delay 3 were more frequently considered within the top 3 most important overall by participants.*

Finally the image quality seems low, this is a consistent problem for the default graphic outputs of Microsoft office applications. Check to be sure exports of images are at least 600 DPI which will require some manual adjustment of these output settings.

This request is at odds with the PLOSone figure resolution requirements (<https://journals.plos.org/plosone/s/figures#loc-resolution>) “Submit figures at the …. with a resolution no greater than **300-600 dpi**…. Resolution below 300 results in blurred, jagged or pixelated published figures. Resolution above 600 dpi may lead to resizing of the published figure.”

We have edited the figures to ensure the DPI resolution falls within this range.
